# Clinical Outcomes of Invasive Home Mechanical Ventilation in Children With Trisomy 13 and Trisomy 18

**DOI:** 10.1002/ppul.71923

**Published:** 2026-11-13

**Authors:** Emmanuel Kumar, Glenn E. Green, Fauziya Hassan, Ann Marie Ramsey, Marc B. Hershenson, Thomas G. Saba

**Affiliations:** ^1^ Department of Pediatrics University of Michigan Medical School Ann Arbor Michigan USA; ^2^ Department of Otolaryngology‐Head and Neck Surgery University of Michigan Medical School Ann Arbor Michigan USA

**Keywords:** Aneuploidy, respiratory failure, ventilator weaning

## Abstract

**Introduction:**

Trisomy 13 and 18 (T13/18) are rare aneuploidies traditionally associated with severe congenital cardiac, pulmonary, neurologic and other anomalies and high 1‐year mortality. Mortality has been improving with greater adoption of life‐sustaining interventions including cardiac surgery, tracheostomy and home mechanical ventilation (HMV) but long‐term outcomes of invasive HMV in this population is not known. The objective of this study was to evaluate outcomes of invasive HMV in T13/18. We hypothesized longer times to HMV liberation among children with T13/18 (Group 1) compared to other children on invasive HMV (Group 2, trisomy 21; Group 3, tracheo‐bronchomalacia without aneuploidy; Group 4, all other causes of respiratory failure without aneuploidy).

**Methods:**

This was a retrospective, descriptive, observational study of electronic medical records. Median duration of mechanical ventilation was compared using non parametric tests. Results: Among 20 patients with T13/18 initiated on invasive HMV, 5 were liberated (25%). All patients liberated also underwent cardiac surgery and gastrostomy tube placement. A greater proportion of patients in Group 2 (68%), Group 3 (66%) and Group 4 (41%) were liberated from HMV. Among patients liberated from invasive HMV in all 4 groups, there was no significant difference in the median duration, in months (IQR), of invasive HMV: Group 1, 25 (15.5–41.5); Group 2, 23 (14–31); Group 3, 21 (16.25–27.75); Group 4, 19 (13–37.5); *p* = 0.928. Failed liberation from HMV in patients with T13/18 was attributed to additional comorbidities including seizures and gastrointestinal disease.

**Conclusion:**

Although the proportion of children with T13/18 liberated from HMV was lower than other groups, the duration of HMV among those who were liberated was comparable to that of children without T13/18, when accompanied by other life‐sustaining interventions.

## 1 Introduction

Trisomy 13 and 18 (T13/18) are rare autosomal aneuploidies associated with significant morbidity and mortality. The reported incidence is approximately 1.15 and 2.63 per 10,000 total births, respectively. In the absence of life‐sustaining interventions—such as cardiac surgery or respiratory support—1‐year mortality approaches 90% [1]. The clinical phenotype is complex and highly variable, with mosaic and partial forms typically associated with milder manifestations [2]. Affected individuals commonly exhibit multi‐system involvement, including orofacial, respiratory, gastrointestinal, cardiac, genitourinary, neurologic, and limb manifestations. Because of high mortality and significant long‐term comorbidities, T13/18 were often considered “lethal” conditions, and care was frequently limited to comfort‐focused or hospice approaches. However, with life‐sustaining interventions, including congenital heart surgery [3], tracheostomy and mechanical ventilation [4], gastrostomy tubes [5], and treatment for hepatoblastoma [6, 7], survival has significantly improved. In response to these evolving outcomes, the American Academy of Pediatrics now recommends an individualized, evidence‐based approach to care, emphasizing shared decision‐making that is informed by current data rather than historical assumptions or bias [8].

Although intensive surveillance and medical interventions have improved survival, individuals with T13/18 continue to experience substantial multi‐organ comorbidities. Pulmonary complications play a critical role in outcomes, contributing to morbidity following cardiac surgery [9] and remaining a leading cause of death [10]. Congenital upper and lower airway abnormalities—including glossoptosis, hypopharyngeal collapse, laryngomalacia, tracheomalacia, and bronchomalacia—are highly prevalent and near‐universal in this population [11]. Sleep‐disordered breathing, particularly obstructive sleep apnea, is estimated to affect ~50% of patients [12]. In addition, central apnea and seizure‐related events frequently contribute to cardiopulmonary arrest and mortality [13].

Invasive home mechanical ventilation (HMV) via tracheostomy is an established life‐sustaining therapy for children with a range of pulmonary and neuromuscular disorders, with utilization increasing globally [14]. The median age at liberation from HMV is typically 23–24 months [15, 16]. This intervention is intended to support gas exchange, facilitate lung and neurocognitive development, and improve quality of life. However, invasive HMV is associated with significant risks, including tracheostomy obstruction and infection, impaired vocalization and swallowing, and a substantial reliance on skilled caregiving, fundamentally altering the home environment and family dynamics. While outcomes and expectations of invasive HMV are relatively well characterized in other pediatric populations—such as bronchopulmonary dysplasia and neuromuscular disease—its use in T13/18 remains poorly defined. In the context of a growing use of life‐sustaining interventions in T13/18, it is essential to determine whether invasive HMV provides meaningful clinical benefit for this population.

The objective of this study was to examine the clinical characteristics and outcomes of patients with T13/18 treated with invasive HMV. We hypothesized that, due to their substantial multi‐organ comorbidities, patients with T13/18 would require longer durations of HMV compared to patients without T13/18 requiring invasive HMV.

## 2 Methods

We conducted a retrospective review of the electronic medical records of all patients enrolled in the University of Michigan Pediatric Ventilator Program between January 1, 2012 and January 1, 2025, with follow‐up through January 15, 2026. All children receiving invasive HMV were included; those managed exclusively with noninvasive ventilation were excluded. The database captured detailed clinical and demographic information including date of birth, gestational age, age at initiation of chronic mechanical ventilation, indication for ventilation, comorbid conditions, underlying aneuploidies including T13/18, trisomy 21 (T21), date of tracheostomy placement, date of liberation from chronic mechanical ventilation, date of decannulation, and corresponding durations of mechanical ventilation and tracheostomy dependence. Race and ethnicity data were based on caregiver reports in electronic medical records. Since the Ventilator Program includes a large patient population with wide clinical variability, patients were stratified into four different groups with unique clinical characteristics: Group 1, T13/18; Group 2, T21; Group 3, tracheo‐bronchomalacia (TBM) without T13/18/21; and Group 4, no TBM or T13/18/21. Group 4 was comprised of patients with a variety of conditions including bronchopulmonary dysplasia (BPD) (21%), chest wall disorders (22%), other congenital pulmonary abnormalities (e.g., hypoplasia) (27%) and neuromuscular disorders (30%).

Children were enrolled in the Pediatric Ventilator Program if they had chronic hypoxemic and/or hypercapnic respiratory failure requiring long‐term invasive HMV. Longitudinal management has been previously described [17] and was guided by evidence‐based clinical practice guidelines [18]. Initiation of invasive HMV was defined as the age at tracheostomy placement in anticipation of long‐term ventilatory support. Liberation from invasive HMV was defined as the age at which a patient maintained adequate oxygenation and ventilation for at least 3 consecutive months without any form of positive pressure ventilation following completion of a standardized institutional weaning protocol [17].

Statistical analyses were performed using GraphPad Prism software, with statistical significance defined as *p* < 0.05. Data are presented as medians with interquartile ranges (IQR). Comparisons of median duration of ventilation across groups were performed using the Kruskal–Wallis test. Kaplan–Meier analyses were used to estimate the proportion of patients remaining on invasive HMV over time. This study was reviewed by the University of Michigan Medical School Institutional Review Board and was determined to be exempt per category 4(iii) at 45 CFR46.104(d) (HUM00217833).

## 3 Results

A total of 304 children with chronic respiratory failure were followed in the Pediatric Ventilator Program during the study period, including 20 with T13/18 (Group 1), 21 with T21 (Group 2), 151 with TBM without T13/18/21 (Group 3), and 111 without TBM or T13/18/21 (Group 4). Nearly all cases of invasive HMV in Groups 1 and 2 were initiated from 2017 to 2025; initiation in Groups 3 and 4 was evenly distributed between 2012 and 2025 (Supplement). Demographics and outcomes of patients in the Pediatric Ventilator Program are described in Figure 1.

**Figure 1:**
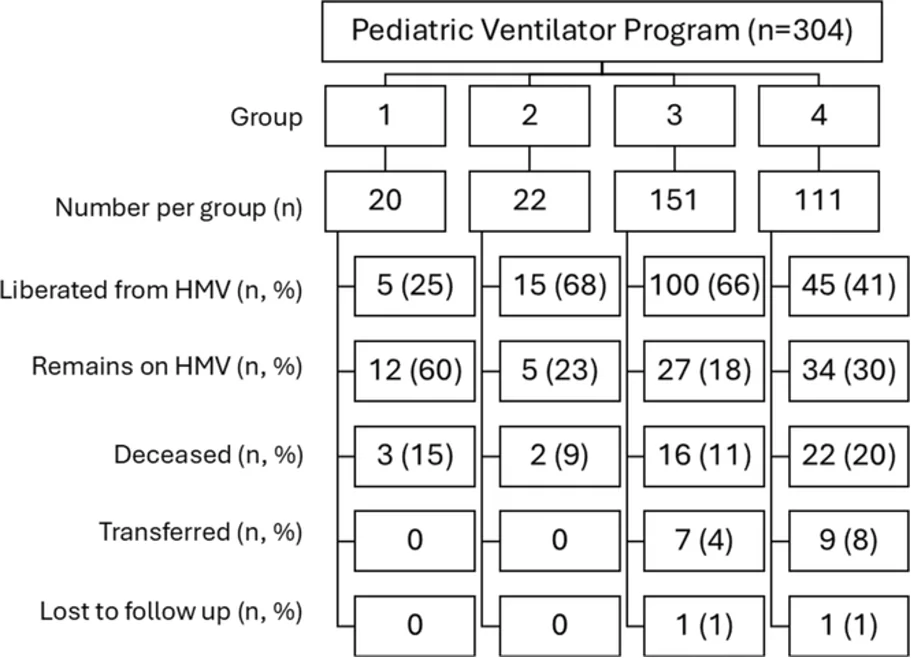
Demographics of patients in the 4 cohorts, including proportion of patients liberated from invasive home mechanical ventilation during the study period. Group 1: trisomy 13 or trisomy 18; Group 2: trisomy 21; Group 3: tracheo‐bronchomalacia and no trisomy; Group 4: all other patients and no trisomy. HMV, home mechanical ventilation.

Among the 20 patients in Group 1, 2 had T13 and 18 had T18 (Tables 1 and 2). All were born between 35 and 41 weeks' gestation. Eighteen were initiated on HMV between 0 and 4 months of age, and two between 6 and 12 months of age. Twelve patients identified as Caucasian, five as African American and three as mixed Caucasian/African American. No patients reported Hispanic ethnicity. Nine were male, and eleven were female. The primary cause of chronic respiratory failure was tracheo‐bronchomalacia in all but 2 patients; central apnea and hypoventilation were the primary cause for the other 2 patients. All patients had congenital heart disease (CHD), and all but three underwent cardiac surgery. Ten (50%) had seizures, and every patient had a gastrostomy tube. Five patients were liberated from HMV, 12 remained HMV‐dependent at the time of analysis, and 3 died while receiving HMV. Of the deaths, two were of unknown cause and one was attributed to a cerebellar herniation. Eighteen patients (90%) survived 1 year of age.

Among the five patients liberated from HMV in Group 1, all had T18 (Table 1). The primary reasons for chronic respiratory failure in these patients were tracheomalacia, bronchomalacia or both in four patients and central sleep apnea (CSA) in one. All five had CHD requiring surgery and all had gastrostomy tubes. Three had a seizure disorder. Only one patient underwent polysomnography prior to liberation, which demonstrated persistent CSA that subsequently improved, leading to successful liberation. Two of the five patients underwent tracheal decannulation, while three remained tracheostomy‐dependent at the time of the analysis.

**Table 1:** Characteristics of patients with Trisomy 13 and Trisomy 18 that were liberated from invasive home mechanical ventilation.

Trisomy	Sex	Age at Ventilation initiation (months)	Age at ventilation liberation (months)	Ventilation duration (months)	Age at tracheal decannulation (months)	Primary cause of chronic respiratory failure	CHD	Cardiac surgery	CSA	Seizures
T18	F	4	20	16	NA	TBM	AVSD	Yes	No	Yes
T18	M	< 1	50	49	90	BM	TOF	Yes	No	Yes
T18	F	10	36	26	NA	CSA	ASD, CoA, PDA, bicuspid aortic valve	Yes	Yes	No
T18	M	2	36	34	NA	TBM	VSD, ASD, left‐sided aortic arch, PDA	Yes	No	Yes
T18	F	3	18	15	27	BM	VSD, ASD	Yes	No	No

Abbreviations: ASD, atrial septal defect; AVSD; atrioventricular septal defect; BM, bronchomalacia; CHD, congenital heart disease; CoA, coarctation of the aorta; CSA, central sleep apnea; PFO, patent foramen ovale; PDA, patent ductus arteriosus; SVC, superior vena cava; TBM, tracheo‐bronchomalacia; TOF, tetralogy of fallot; T13, Trisomy 13; T18, Trisomy 18; VSD, ventricular septal defect

The 12 patients who remained dependent on HMV had variable ventilatory requirements, ranging from continuous support (24 h per day) to nocturnal‐only ventilation (Table 2). This group included 2 patients with T13 and 10 with T18. The underlying etiologies of respiratory failure leading to initiation of HMV was tracheomalacia, bronchomalacia or both (*n* = 11), and hypoventilation (*n* = 1). Ongoing HMV dependence and failed liberation was attributed to a combination of seizures, recurrent respiratory illnesses, gastrointestinal comorbidities, and CSA. Three patients demonstrated successful weaning progress but required a modified weaning strategy including smaller setting changes and longer periods at every weaning stage. All had CHD, and 11 underwent surgery. Seven patients underwent polysomnography, with CSA identified in one. Seven wpatients were on medications for seizure disorders.

**Table 2:** Characteristics of patients with Trisomy 13 and Trisomy 18 that were not liberated from home mechanical ventilation at the time of the study.

Trisomy	Sex	Age at Ventilation initiation (months)	Age at ventilation duration (months)	Primary cause of respiratory failure	CHD	Cardiac surgery	CSA	Seizures	Deceased on vent	Factors contributing to continued ventilation
T13	F	2	29	TBM	ASD, VSD, CoA	Yes	No	Yes	No	Seizures
T13	M	3	27	BM	Mitral Valve Prolapse and regurgitation, L aortic arch with aberrant right subclavian artery	No	No	Yes	No	Frequent illnesses
T18	M	2	NA	TBM	VSD, PDA, ASD	No	No	Yes	Yes	NA
T18	F	5	88	TBM	PFO, VSD, PDA	Yes	No	No	No	Frequent illnesses
T18	F	0	69	TBM	VSD, bicuspid aortic valve, PDA, PFO	No	No	No	No	Weaning intolerance
T18	M	1	62	TBM	VSD, ASD, pulmonary stenosis, PDA	Yes	No	No	No	Gastrointestinal comorbidities
T18	F	1	63	TBM	VSD, pulmonary valve stenosis, PFO, ASD	Yes	No	No	No	Seizures
T18	M	1	55	TBM	AVSD, bicuspid aortic valve, bicuspid pulmonary valve	Yes	No	Yes	No	Frequent illnesses, seizures
T18	M	0	NA	TBM	PDA, VSD	Yes	No	No	Yes	NA
T18	F	0	37	TBM	PFO, VSD, Bilateral SVC	Yes	No	No	No	Frequent illnesses, weaning intolerance
T18	M	1	NA	TBM	VSD, Coarctation of the Aorta	Yes	No	No	Yes	NA
T18	M	0	26	TM	VSD, Aortic Arch Hypoplasia, RIght Aberrant Subclavian Artery, Bicuspid aortic valve	Yes	No	No	No	Gastrointestinal comorbidities
T18	F	13	20	BM	VSD	Yes	No	Yes	No	Seizures
T18	F	3	23	TBM	VSD	Yes	Yes	Yes	No	CSA
T18	F	1	23	HV	Mitral Valve stenosis, VSD	Yes	No	Yes	No	Weaning intolerance

Abbreviations: ASD, atrial septal defect; AVSD; atrioventricular septal defect; BM, bronchomalacia; CHD, congenital heart disease; CoA, coarctation of the aorta; CSA, central sleep apnea; HV, hypoventilation; PFO, patent foramen ovale; PDA, patent ductus arteriosus; SVC, superior vena cava; TBM, tracheobronchomalacia; TM, tracheomalacia; TOF, tetralogy of fallot; T13, Trisomy 13; T18, Trisomy 18; VSD, ventricular septal defect

We compared the outcomes of children with T13/T18 to other groups. The median age of tracheostomy placement, in months, was 1.5 (IQR 1–3) in Group 1, 4 (2–5.25) in Group 2, 3 (2–6) in Group 3 and 6 (3–86) in Group 4. Among patients in the Pediatric Ventilator Program who were not deceased or lost to follow‐up, liberation from HMV was achieved in 5 of 17 patients in Group 1 (29%), 15 of 20 in Group 2 (75%), 100 of 127 in Group 3 (79%), and 45 of 79 in Group 4 (57%). Among those in Group 4, 20 of 24 (83%) patients with BPD and 25 of 55 (45%) patients without BPD (including those with chest wall abnormalities, congenital pulmonary abnormalities and neuromuscular disease) were liberated from the ventilator. The median duration of HMV, among those liberated from HMV, from initiation to liberation, was 25 months (IQR 15.5–41.5) in Group 1, 23 months (14–31) in Group 2, 21 months (16.25–27.75) in Group 3, and 19 months (13–37.5) in Group 4 (*p* = 0.928) (Figure 2). When patients in Group 4 were stratified into those with BPD and those without BPD, median duration of HMV, in months, was 19 (IQR 13.25,−27) and 20 (IQR 10.5–38.5), respectively. There was no significant difference when compared to Group 1, 2 and 3 (*p* = 0.98). At the time of analysis, excluding those that were deceased or lost to follow up, 12 of 17 patients in Group 1 (71%), 5 of 10 in Group 2 (25%), 27 of 127 in Group 3 (21%), and 34 of 79 in Group 4 (43%) remained dependent on HMV. Kaplan–Meier analysis demonstrated similar proportions of patients liberated from HMV across all groups within the first 16 months after initiation (Figure 3). Beyond this time point, however, rates diverged, with patients in Group 1 liberating at a slower rate compared to other groups. Together, these data demonstrate that while some children with T13/T18 may successfully be liberated from the ventilator, many have delayed weaning compared to other groups.

**Figure 2:**
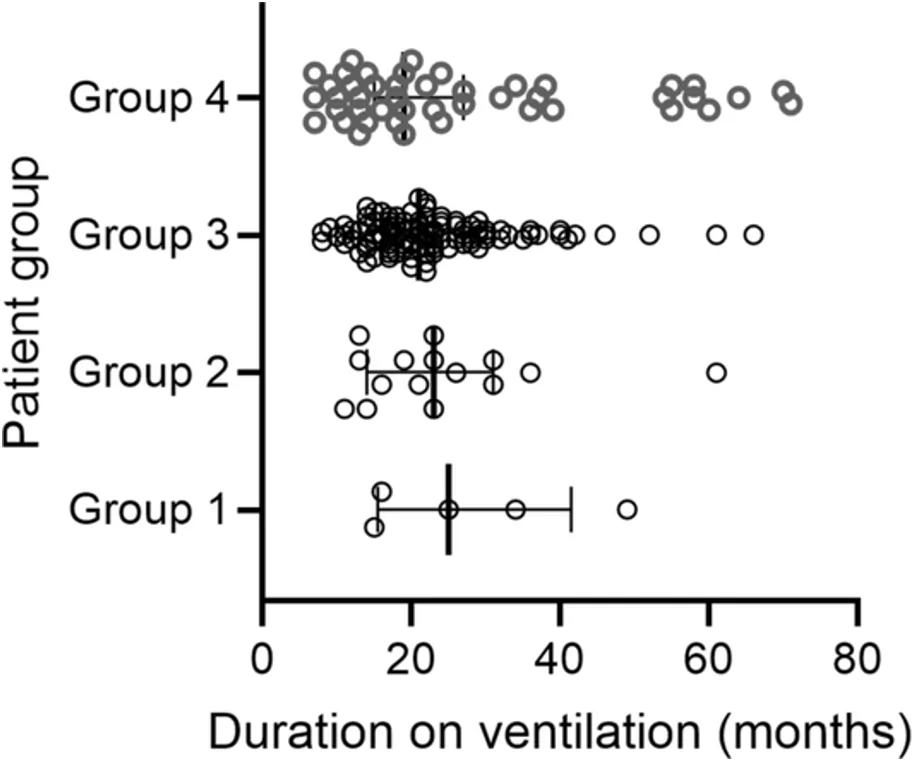
Duration of invasive home mechanical ventilation from tracheostomy to liberation. Each patient is represented by a single circle. Medians in each group are represented by a vertical line with interquartile range. Group 1: trisomy 13 or trisomy 18; Group 2: trisomy 21; Group 3: tracheo‐bronchomalacia and no trisomy; Group 4: all other patients and no trisomy.

**Figure 3:**
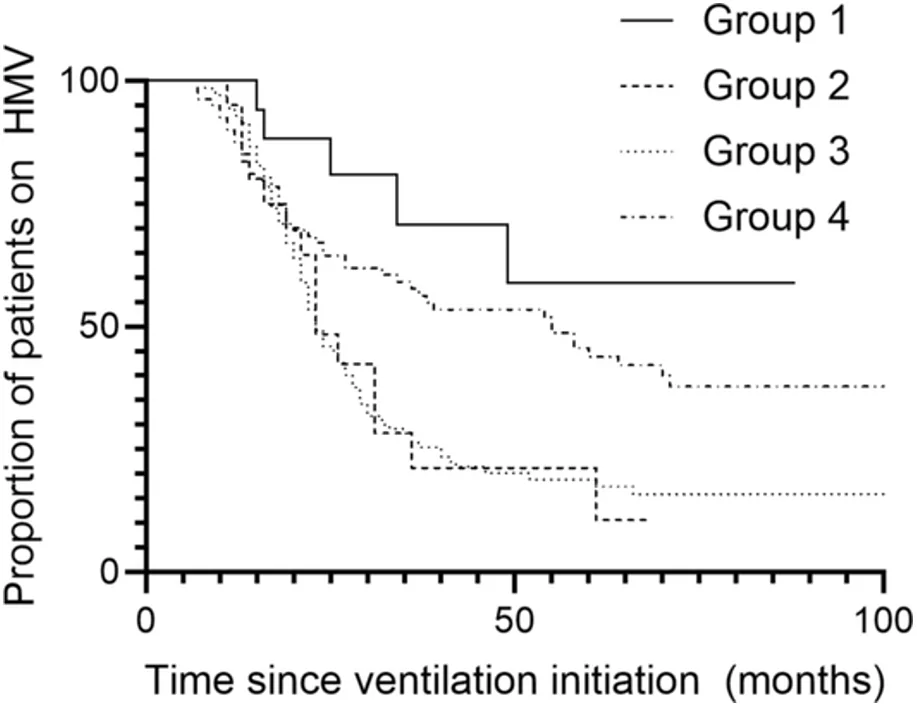
Percent of patients remaining dependent on home mechanical ventilation over time since initiation. HMV, home mechanical ventilation. Group 1: trisomy 13 or trisomy 18; Group 2: trisomy 21; Group 3: tracheo‐bronchomalacia and no trisomy; Group 4: all other patients and no trisomy.

## 4 Discussion

In this study, we describe a single‐center experience with invasive HMV in 20 patients with T13/18 compared to other patients requiring invasive HMV. Among 20 patients with T13/18, 5 patients with T18 were successfully liberated from invasive HMV using standard institutional protocols of the Pediatric Ventilator Program.

To our knowledge, this is the first study to demonstrate that, despite significant comorbidity burden, selected patients with T13/18—particularly in the context of other life‐sustaining interventions such as cardiac surgery—can be successfully supported on HMV until underlying contributors to respiratory failure, including tracheo‐bronchomalacia, improve. Contrary to our initial hypothesis, although patients with T13/18 received tracheostomies relatively earlier, patients with T13/18 who achieved HMV liberation did not experience longer durations of HMV compared to patients with T21, tracheo‐bronchomalaica or other causes of chronic respiratory failure. The median duration of HMV in T13/18 was comparable across groups. The proportion of patients liberated from HMV was lowest in the T13/18 group, particularly after the first 16 months of HMV. Long‐term outcomes of the patients with T13/18 remaining dependent on HMV are unclear.

Twelve patients with T13/18 remained ventilator‐dependent at the time of analysis due to multifactorial comorbidities including seizures, gastrointestinal comorbidities, recurrent respiratory illness, and intolerance of standard institutional weaning protocols. Although central sleep apnea is highly prevalent in T13/18 [19], it was not a major contributor to delayed liberation in this cohort. Rather, delayed liberation appears to reflect the cumulative impact of multiple comorbid conditions, underscoring the need for individualized, flexible weaning strategies. Variability in duration of HMV among patients in different groups who were not liberated likely reflects underlying differences in disease pathophysiology across groups. Notably, many patients without T13/18/21 (Group 4) had neuromuscular disorders in which long‐term or lifelong ventilatory support is expected. A formal comparison of HMV outcomes between groups, therefore, is limited by variability in the underlying cause of respiratory failure. While a select subset of children with T18 were liberated from HMV, it is important to acknowledge that a majority did not, likely due to the multisystem comorbidities characteristic of T18.

Although data on HMV outcomes in T13/18 remain limited, a growing body of literature supports improved survival with the use of life‐sustaining interventions. In one single‐center cohort of 72 newborns with T13/18, 1‐year survival was 70% among those receiving intensive interventions compared with 20% among those managed with comfort care [20]. Similarly, population‐level data demonstrate increasing survival over time alongside greater use of interventions such as cardiac surgery and tracheostomy [21, 22]. In our cohort, 1‐year survival reached 90% among children with T13/18 receiving comprehensive care, including HMV and cardiac surgery. Together, these findings contribute to evolving evidence that selected patients with T13/18 can achieve meaningful survival with appropriately tailored, multidisciplinary care.

The American Academy of Pediatrics emphasizes that management decisions for children with T13/18 should be individualized and grounded in shared decision‐making, without reliance on historical assumptions regarding prognosis [8]. At our institution, care for children with T13/18 and chronic respiratory failure is delivered through a coordinated Pediatric Ventilator Program (including 304 patients) and Trisomy Clinical Program (including 67 patients with T18 and 16 patients with T13), supported by experienced multidisciplinary teams and evidence‐based guidelines. This integrated approach facilitates proactive identification and management of comorbidities, frequent longitudinal follow‐up, and informed, collaborative decision‐making with families. Accordingly, the provision of life‐sustaining interventions, including HMV, is best undertaken within structured and experienced clinical programs.

This study has several limitations. The sample size of patients with T13/18 was small, and group sizes were uneven, resulting in a marked reduction in statistical power. Only invasive HMV was evaluated; patients initiated on non‐invasive positive pressure ventilation (NIPPV) for chronic respiratory failure were not included in the study although some patients in Groups 3 and 4 used NIPPV as part of the weaning process towards liberation. All patients with T13/18 in this cohort were infants for whom long term NIPPV at home was not feasible. Whereas many patients with T13/18 receive NIPPV as part of management of acute or chronic respiratory failure, modes, strategies, and interfaces vary considerably, and only invasive HMV was evaluated in this study. Ventilator modes and settings were not analyzed in detail, although all patients were managed according to standardized institutional program guidelines. Since invasive HMV is a relatively novel approach to children with T13/18, follow‐up time is relatively short compared to other patient groups in the Pediatric Ventilator Program, and long‐term outcomes remain unclear. Age at initiation of HMV was defined by the date of tracheostomy placement, although many patients began ventilatory support prior to tracheostomy. This limitation likely affected all groups equally. Tracheostomy decannulation outcomes were not systematically evaluated due to the small number of tracheal decannulation events in the T13/18 group. Future studies should examine rates and timing of decannulation, as well as the natural history and outcomes of congenital airway abnormalities, which are highly prevalent yet incompletely understood in this population [11].

In this cohort, 20 children with T13/18 received invasive HMV, with 90% surviving to 1 year. Five patients were successfully liberated from HMV at a median of 25 months, and the duration of support was comparable to that observed in patients without T13/18. To our knowledge, this is the first study to evaluate HMV outcomes in this population. These findings demonstrate that, within structured clinical programs—combining a dedicated ventilator program, trisomy‐focused multidisciplinary care, evidence‐based management and weaning protocols, and adjunctive life‐sustaining interventions such as cardiac surgery and gastrostomy—selected children with T13/18 can be successfully supported on and liberated from invasive HMV. Collaboration with a multi‐disciplinary, evidence‐guided medical team, including palliative care, and a shared‐medical decision‐making approach, respecting family values, are essential for difficult medical decision‐making, including the decision to pursue tracheostomy. An assessment of the family decision‐making process was out of the scope of this study and would be an opportunity for future research.

## Author Contributions

**Emmanuel Kumar:** conceptualization, investigation, writing – original draft, methodology, writing – review and editing, formal analysis, data curation. **Glenn E. Green:** conceptualization, formal analysis. **Fauziya Hassan:** conceptualization, formal analysis. **Ann Marie Ramsey:** conceptualization, formal analysis. **Marc B. Hershenson:** conceptualization, investigation, methodology, writing – review and editing. **Thomas G Saba:** conceptualization, investigation, writing – original draft, methodology, writing – review and editing, formal analysis, project administration, data curation, supervision, resources.

## Funding

The authors have nothing to report.

## Ethics Statement

This study was reviewed by the University of Michigan Medical School Institutional Review Board and was determined to be exempt per category 4(iii) at 45 CFR46.104(d): secondary research for which consent is not required (HUM00217833).

## Conflicts of Interest

Glenn E Green has a patent for an implantable airway splint.

## Supporting information


Supporting File

## Data Availability

The data that support the findings of this study are available on request from the corresponding author. The data are not publicly available due to privacy or ethical restrictions.
